# Antimicrobial Activity of Cationic Poly(3-hexylthiophene) Nanoparticles Coupled with Dual Fluorescent and Electrochemical Sensing: Theragnostic Prospect

**DOI:** 10.3390/s21051715

**Published:** 2021-03-02

**Authors:** Nada Elgiddawy, Shiwei Ren, Wadih Ghattas, Waleed M. A. El Rouby, Ahmed O. El-Gendy, Ahmed A. Farghali, Abderrahim Yassar, Hafsa Korri-Youssoufi

**Affiliations:** 1 Université Paris-Saclay, CNRS, Institut de Chimie Moléculaire et des Matériaux d’Orsay (ICMMO), ECBB, 91400 Orsay, France; n.giddawy@psas.bsu.edu.eg (N.E.); wadih.ghattas@universite-paris-saclay.fr (W.G.); 2 Department of Biotechnology and Life Sciences, Faculty of Postgraduate Studies for Advanced Sciences (PSAS), Beni-Suef University, Beni-Suef 62 511, Egypt; 3 LPICM, CNRS, Ecole Polytechnique, Institut Polytechnique de Paris, Route de Saclay, 91128 Palaiseau, France; shiwei.ren@polytechnique.edu (S.R.); abderrahim.yassar@polytechnique.edu (A.Y.); 4 Materials Science and Nanotechnology Department, Faculty of Postgraduate Studies for Advanced Sciences (PSAS), Beni-Suef University, Beni-Suef 62 511, Egypt; waleedmohamedali@psas.bsu.edu.eg (W.M.A.E.R.); farghali@psas.bsu.edu.eg (A.A.F.); 5 Microbiology and Immunology Department, Faculty of Pharmacy, Beni-Suef University, Beni-Suef 62511, Egypt; ahmed.elgendy@pharm.bsu.edu.eg; 6 Laser Institute for Research and Applications LIRA, Beni-Suef University, Beni-Suef 62511, Egypt

**Keywords:** cationic conjugated-polymers, nanoparticles, mini-emulsion, fluorescence, impedance, detection, bacteria, fungi, antimicrobial, biocompatibility

## Abstract

Designing therapeutic and sensor materials to diagnose and eliminate bacterial infections remains a significant challenge for active theragnostic nanoprobes. In the present work, fluorescent/electroactive poly(3-hexylthiophene) P3HT nanoparticles (NPs) stabilized with quaternary ammonium salts using cetyltrimethylammonium bromide (CTAB), (CTAB-P3HT NPs) were prepared using a simple mini-emulsion method. The morphology, spectroscopic properties and electronic properties of CTAB-P3HT NPs were characterized by DLS, zeta potential, SEM, TEM, UV-vis spectrophotometry, fluorescence spectroscopy and electrochemical impedance spectroscopy (EIS). In an aqueous solution, CTAB-P3HT NPs were revealed to be uniformly sized, highly fluorescent and present a highly positively charged NP surface with good electroactivity. Dual detection was demonstrated as the binding of the bacteria to NPs could be observed by fluorescence quenching as well as by the changes in EIS. Binding of *E. coli* to CTAB-P3HT NPs was demonstrated and LODs of 5 CFU/mL and 250 CFU/mL were obtained by relying on the fluorescence spectroscopy and EIS, respectively. The antimicrobial activity of CTAB-P3HT NPs on bacteria and fungi was also studied under dark and nutritive conditions. An MIC and an MBC of 2.5 µg/mL were obtained with *E. coli* and with *S. aureus*, and of 0.312 µg/mL with *C. albicans*. Additionally a good biocompatibility toward normal human cells (WI38) was observed, which opens the way to their possible use as a therapeutic agent.

## 1. Introduction

Concerns related to bacterial infections are often raised due to major outbreaks that have a high overall mortality rate [1]. Moreover, the emergence of drug-resistant bacterial strains as a cause of intensive use of antibiotics makes bacterial infections a major global health crisis [2,3,4]. Thus, the timely diagnosis and efficient alternative antimicrobial treatment of pathogenic infections are of great importance in dealing with such a crisis [5]. To address this issue, many researcher efforts’ have been devoted to the design the next generation of detection probes with antimicrobial activity, serving both diagnostic and therapeutic purposes at one time (theragnostic antimicrobial) [5,6].

In this regard, fluorescence sensing is a promising approach for quick and easy medical diagnosis. This is because of the availability of many fluorescent probes with easy readout, non-destructive operation, rapid signal generation and sensitive detection [7,8]. Besides their use as fluorescent probes, many advanced fluorescent materials have been reported as smart multi-tasking probes for biomedical applications including bacterial identification, discrimination and killing.

Additionally, there are many reports on the antibacterial effects of various nano-structured materials and nanoparticles (NPs), including antibiotics coated NPs [9], Ag-coated Au NPs [10], antimicrobial peptides conjugated with Gd^3+^ [11] and hydrogels [12]. Among the fluorescent materials, conjugated polymer nanoparticles (CPNs) have emerged as excellent probes for fluorescence imaging, photoacoustic imaging and photothermal therapy for microbial infections [13] and cancer [14]. The great interest in using CPNs is motivated by their outstanding photophysical properties, excellent photostability, high quantum efficiency, super sensitivity and amplified fluorescent quenching effect [15,16,17] with high biocompatibility and biodegradability [18]. In biosensing and biomedical applications CPNs need to be functionalized for subsequent bioconjugation. Such functionalization may be achieved by following two approaches. The first approach is the direct functionalization of conjugated polymers which involves covalent grafting of functional groups to monomers followed by polymerization, and the post-polymerization functionalization strategy [19]. The second approach is an affinity-driven binding of secondary capping layers which relies on interactions, usually hydrophobic, between the conjugated chains and a secondary capping agent [20]. For example Wu et al. reported CPNs modified by amphiphilic polymers such as polyethylene glycol and poly (styrene maleic anhydride) that were adsorbed on the surface of NPs to increase hydrophilicity and to anchor sites for subsequent use for biosensing, imaging and therapy [21].

Cationic conjugated polymers (CCPs) have been extensively used as a novel antibacterial material with remarkable activity and are considered promising candidates to overcome bacterial resistance [22,23,24,25]. These cationic polymers have a broad spectrum of activity, killing or inhibiting the growth of both wild-type and antibiotic-resistant bacteria with remarkable efficacy by two different modes of activity: under light illumination and in the dark. The dark mode activity includes membrane disruption and the light-activated mode activity involves the generation of reactive oxygen species (ROS) [26,27,28]. Thus, cationic functional groups and π-conjugated hydrophobic systems of CCPs are both essential for antimicrobial performances [28]. Furthermore, the ability of CCPs to bind to the membrane of bacteria by electrostatic and hydrophobic interactions has been explored as a driving force for the detection of pathogens. Yuan et al. developed a CCP, poly(phenylene vinylene) (PPVNMe3+) derivative which displayed specific interactions with different components of microbial cell envelopes. They demonstrated that only through varying the ion strengths of the buffer solution a single chain of the polymer could discriminate fungi, Gram-positive and Gram-negative bacteria by using fluorescence measurements. Isothermal titration microcalorimetry and zeta potential measurement suggested different interaction mechanisms including electrostatic and hydrophobic interactions [29].

In the last few years, biocidal poly(thiophene) functionalized with quaternary ammonium groups via flexible alkyl chains have gained much attention. This is due to their ability to strongly absorb visible light and to their outstanding fluorescence properties with a high yield of triplet state formation, and consequently a high yield of singlet oxygen and ROS. Yuang et al. reported an imidazolium-functionalized poly(hexylthiophene) which exhibited remarkably high biocidal efficiency to both Gram-positive and Gram-negative bacteria at sub-μg/mL concentrations while leaving mammalian cells unaffected. This polymer was shown able to produce ROS which endowed it with the ability to inactivate bacteria under visible light [30]. Another group reported the effect of the molecular weight and the nature of the functional group of cationic poly(3-hexylthiophene) polymer on killing various bacteria. The biocidal activity was investigated both in the dark and under irradiation and showed high rates of cell death after photoactivation at polymer concentrations of 10 µg/mL for both gram-positive and gram-negative bacteria *(E. coli* and *B. atrophaeus)*. The effect of the molecular weight of the polymer is minimal. However, the functional groups’ effect on the bacteria membrane, and polymer modified with tertiary amine, demonstrated more damage to bacteria [31]. The same group developed a series of antimicrobial materials with positive charge including modified P3HT and evaluates the speed of the antibacterial activity against a panel of laboratory strains of *Pseudomonas aeruginosa*, *Staphylococcus aureus*, and *Staphylococcus epidermidis* in the dark and upon irradiation [32]. They demonstrate that the modified P3HT polymers, at 10 μg/mL and during 10 min, kill a panel of all the pathogenic bacteria with more than four log reductions in the concentration in the dark and under UV irradiation.

**Scheme 1:**
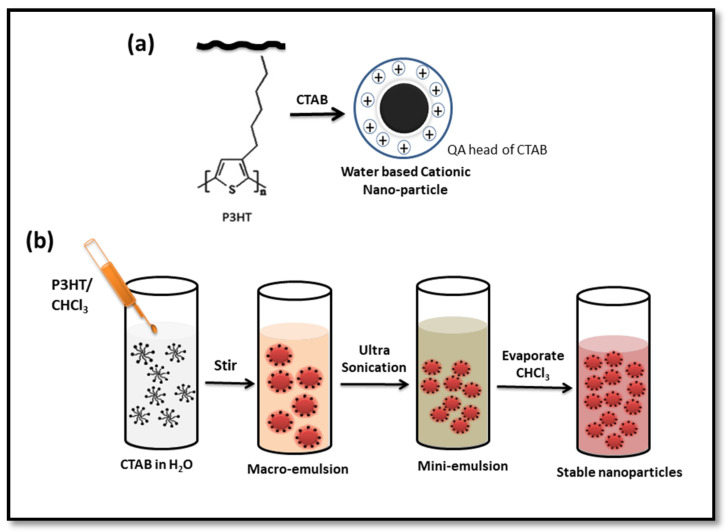
Schematic illustration of nanoparticle formation for CTAB-P3HT (**a**), Scheme for synthesis of nanoparticles by mini-emulsion process (**b**).

Poly(3-hexylthiophene) (P3HT) is the most studied and commercially available conjugated polymer due to its good electrical conductivity, chemical stability, low redox potential and its optical properties in the semiconducting state [33,34]. Despite its vast potential applications in the field of bioelectronics, there are no reports available on its biocompatibility and cytotoxicity. Most of the reported studies on P3HT have been on the chemical modification of the conjugate backbone with positively charged groups. However, these chemical modifications destroy some of its intrinsic properties. In this work, cationic fluorescent cetyltrimethylammonium bromide (CTAB) P3HT NPs (CTAB-P3HT NPs) were prepared and their antimicrobial properties and toxicity were investigated. CTAB-P3HT NPs were produced by a mini-emulsion method, where the π-conjugated P3HT system forms the core by hydrophobic interactions, and the CTAB groups wrapped around the conjugated core to form a positively charged shell layer on the nanoparticle’s surface (Scheme 1). The CTAB groups on the NP facilitate the binding to anionic bacterial cell membranes by electrostatic interactions, thus causing fluorescence quenching. Electrochemical studies were performed to support and corroborate the fluorescence results. Cellular viability experiments were carried out to assess the cytotoxicity effect of CTAB-P3HT NPs. To the best of our knowledge, this is the first time that this simple technique was used for the production of cationic conjugated polymer nanoparticles (CCPNs) with a highly promising dual diagnostic and theragnostic NPs-tool.

## 2. Materials and Methods

### 2.1. Materials

P3HT (MW = 22 kDa D = 2.13 and RR = 90–93%), Cetyltrimethylammonium bromide (CTAB) and chloroform were purchased from Sigma-Aldrich. Nutrient agar, LB broth, agar-agar, peptone, yeast extract and glucose were supplied by HiMedia. *Escherichia coli*, *Staphylococcus aureus* and *Candida albicans* were obtained from the microbiology lab, Beni-Suef University. WI-38 cell line was purchased from American Type Culture Collection (ATCC). Dulbecco’s Modified Eagle’s medium (DMEM) was purchased from Invitrogen/Life Technologies, Fetal bovine serum (FBS) from Hyclone, insulin and penicillin-streptomycin from Sigma-Aldrich.

### 2.2. Preparation of P3HT-NPs

CTAB-P3HT NPs were formed in water by the mini-emulsion method using CTAB as the cationic surfactant. In a typical synthesis, 5 mg P3HT were dissolved in 1 mL of chloroform (Solution 1). The obtained bright orange Solution 1 was slightly warmed to ensure the complete solubility of the P3HT polymer. CTAB solution was prepared in its critical micelle concentration (CMC) (1 mM = 0.36 mg/mL) using purified water, then warmed and sonicated to ensure complete solubility (Solution 2). 250 µL of Solution 1 was immediately injected into 5 mL of Solution 2 to create an oil-in-water emulsion system. The emulsion was stirred at 1000 rpm for 60 min at room temperature forming a macro-emulsion. It was then transferred to an ultrasonic bath and sonicated for 60 min at room temperature to form a mini-emulsion. The mini-emulsion was stirred (1000 rpm) at 70 °C for 30 min to remove solvents. Anionic P3HT nanoparticles were synthesized in a similar manner using an anionic surfactant, sodium dodecyl sulfate (SDS) at its CMC of 8.3 mM.

### 2.3. Bacterial Cell Biosensing Assay

#### 2.3.1. Fluorescence Detection

For fluorescence detection, bacterial cells (*E. coli*) were cultured in LB broth, collected by centrifugation and washed with a PBS buffer solution pH 7.2 three times to rinse away any residual medium. Subsequently, bacterial cells concentrations were calibrated using a spectrophotometer with optical density of 1 at OD 600 nm, then diluted to the concentration ranged between 10^1^ to 10^7^ CFU/mL (CFU, colony-forming units). A 100 μL of *E. coli* suspension with different serial dilution concentrations ranged from 10^1^ to 10^7^ CFU/mL were added to 100 μL CCP in the final volume of 200 μL (Final concentration of CTAB-P3HT NPs was 5 µg/mL). The sample was incubated in the dark at room temperature, and then fluorescence spectra were measured. To verify the reproducibility, each measurement was repeated three times on the same day with the same suspension of bacteria. The optimum excitation wavelength was found to be 500 nm with an emission range between 550 and 850 nm. The optical cell was maintained at 22 °C during the measurement.

#### 2.3.2. Electrochemical Detection

CTAB-P3HT NPs modified electrodes were prepared by drop-casting 8 μL of a solution of CTAB-P3HT NPs on a glassy carbon electrode (GCE). The electrodes were dried at 50 °C for 2 h. Modified CTAB-P3HT /electrodes were incubated with various concentrations of bacteria from 10^2^ to 10^7^ CFU/mL at room temperature for 1 h. Detection of *E. coli* was carried out through EIS measurements at −0.4 V using an alternating potential of 10 mV of amplitude in the frequency range of 0.1 Hz to 100 kHz in a sterilized 10 mM PBS buffer solution at pH 7.2, as a label-free electrolyte.

### 2.4. Antimicrobial Activity of CTAB-P3HT NPs

Dynamic interaction with microorganisms, minimum inhibitory concentration (MIC), minimum bactericidal concentration (MBC), minimum fungicidal concentration (MFC), and time-killing assay were assessed to evaluate the antimicrobial activity of CTAB-P3HT NPs. *E. coli* and *S. aureus* were selected as representative Gram-negative and Gram-positive bacterial models for the antibacterial assays and *C. albicans* for the antifungal activity.

#### 2.4.1. Minimum Inhibitory Concentration (MIC)

MIC was determined using the broth macro dilution technique [35]. LB broth was used for bacteria and Peptone Yeast extract Dextrose (PYD) broth was used for fungi. Concisely, different concentrations (0.312, 0.625, 1.25, 2.5, 5, 10, 15 and 20 μg/mL) of synthesized CTAB-P3HT NPs were prepared in broth. A 20 μL overnight culture of each tested microorganism was added to 2 mL of the nutrient broth to obtain starting inoculums of approximately 4–6 × 10^6^ CFU/mL. A positive tested control which included the microorganisms and the nutrients and a negative control which consisted of the nutrients broth were used as references. MIC was defined as the minimum concentration of the tested material that achieves complete microbial inhibition compared with the positive control after incubation for 24 h at 37 °C.

#### 2.4.2. MBC and MFC Determination

Minimal bactericidal and fungicidal concentrations (MBC and MFC) were determined according to Lemos et al. [36]. Briefly, 0.1 mL from all clear MIC wells (no growth seen in macro-dilution trays) were transferred onto LB agar plates for bacteria and PYD agar plates for fungi. MBCs and MFCs were evaluated by plate colonies counts corresponding to the total viable cells.

#### 2.4.3. Time-Killing Assay

A time-killing test is used to study the dynamic interaction between the antimicrobial agent and the microbial strain [36,37]. For bacteria, this test has been well standardized and described in the M26-A document of the Clinical and Laboratory Standard Institute (CLSI) [38]. It was performed in a broth culture medium using three tubes containing either 5 × 10^5^ CFU/mL of bacterial suspension or 5 × 10^4^ CFU/mL of fungal suspension. The first and second tubes were used to test CTAB-P3HT at the final concentration of 0.25 MIC and 1 MIC compared to the normal growth behavior at the third tube (positive control). The incubation was done under suitable conditions and the number of cells was measured at time intervals (0, 1, 2, 3, 4, 6 and 8 h) for bacteria and (0, 1, 2, 3, 4, 6 8, 12 and 20 h) for fungi. The number of total living cells (CFU/mL) of each tube at different times was calculated using the agar plate count method.

#### 2.4.4. Cytotoxicity to Mammalian Cells

The WI-38 cell line was cultured using DMEM supplemented with 10% FBS, 10 µg/mL of insulin and 1% penicillin-streptomycin in 100 µL in a 96-well plate reaching a cell density of 1.2–1.8 × 10,000 cells/well. Cells were placed in the presence of 100 µL of an aqueous solution of CTAB-P3HT NPs per well for 48 h at 37 °C before toxicity evaluation by the MTT assay. The absorbance for MTT analysis was evaluated at a wavelength of 570 nm. The background absorbance of multi-well plates was also measured at 690 nm and subtracted from the 450 nm measurement.

### 2.5. Instrumentation

The average size of NPs was measured in 18 megohm-cm deionized water by dynamic light scattering (DLS). Zeta potentials and size measurements were performed at 25 °C using a Zetasizer Nano ZS 90 from Malvern Zies sigma 500. The morphology of the synthesized CTAB-P3HT NPs was recorded using a Field emission scanning electron microscope (FE-SEM) with JEOL JEM-2100 and an internal charge coupled device (CCD) camera. High resolution Transmission electron microscope (HR-TEM). Ultraviolet-visible (UV-vis) absorption spectra were carried out on a Carry 300 Bio spectrophotometer. Fluorescence measurements were taken on a spectrophotometer TECAN infinite M200 Pro. The fluorescence images were taken by fluorescence microscopy Leica DMi8, RHOD LP excitation 540/45 nm, dichroic 580 nm, emission LP590 nm. Magnification of the object lens was 40×. FT-IR measurement was performed with Bruker Vertex 70 equipped with ATR pike and MCT detector. NMR spectra was collected on a Bruker ARX 400 NMR spectrometer. EIS measurements were carried out in 10 mM PBS buffer solution pH 7.2 using a Metrohm Autolab PGSTAT12 Potentiostat. A three-electrode system was used with a Pt wire as the counter electrode, Ag/AgCl as a reference electrode and GCE as a working electrode. For MTT assay measurements, microplate Bioline ELISA reader was used.

## 3. Results and Discussion

### 3.1. Preparation of CTAB-P3HT NPs

The mini-emulsion method is a versatile approach to produce stable, water-dispersible nanoparticles [39,40]. In such NPs, the polymer chains are typically wrapped by a shell of surfactant, which allows their controlled dispersion in an aqueous environment. CTAB-stabilized CPNs possess a positive surface charge due to the cationic head groups facing the solvent [41]. In such NPs, the hydrophobic chains of the polymers are entwined with the hydrophobic chains of CTAB to form the nucleus, and the hydrophilic quaternary ammonium groups form shells on the surface (Scheme 1a). The preparation of CCPNs by mini-emulsion is outlined in (Scheme 1b). In a typical preparation procedure P3HT dissolved in chloroform (oil phase) was injected in the aqueous phase in the presence of the CTAB at its CMC, followed by ultrasonication and finally removal of the organic solvent by heating, which could be followed by observing the color change from orange to red-pink [40]. CTAB functional groups wrapped on the surface of nanoparticles provide positive charges to bind and detect bacteria or cells via electrostatic interaction. Additionally, the combination with the hydrophobic character of P3HT enhances the antimicrobial effect against pathogens.

### 3.2. Characterization of CTAB-P3HT NPs

#### 3.2.1. Morphological Characterization

**Figure 1:**
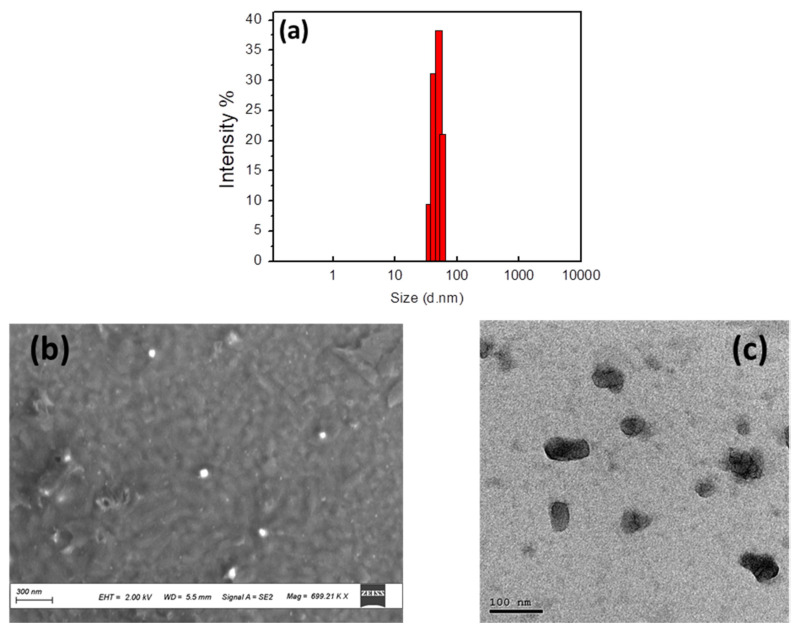
Size distribution intensity (**a**), SEM image (**b**), and TEM images (**c**) of CTAB-P3HT NPs.

The size and size-dispersity of the NPs were determined using DLS in an aqueous solution. The DLS analysis (Figure 1a) shows that P3HT NPs have an average diameter of 55.32 ± 7.3 nm and exhibit monomodal distributions, with a particle dispersion index (PDI) of 0.4. The morphology of CTAB-P3HT NPs was also investigated by SEM and TEM imaging. As shown in SEM images (Figure 1b), CTAB-P3HT NPs appear as spherical particles with a uniform shape which affirms the formation of a stable water-based NP dispersion with no aggregation. TEM images of CTAB-P3HT NPs (Figure 1c) also display uniform and spherical morphology. The particle size and size distribution in TEM are consistent with the DLS data. These results are in good agreement with those reported in the literature for the synthesis of P3HT NPs through the mini-emulsion process, where the oil phase and surfactant influenced the nature of P3HT aggregates. Therefore, the polymer chain rigidity and chain length had a role in the obtained sizes and shapes. The rigidity could be related to the persistence length of the polymer, since with a chain length shorter than the persistence length, the polymer mainly behaves as a rod, while longer chains and higher molecular weights allow more bending and give more spherical shapes [42]. Zeta potential measurements (Table 1) show that CTAB-P3HT NPs have a positive charge of 44.1 ± 5.07 mV that confirms the presence of the CTAB on the surface of the NPs [41]. The P3HT is a hydrophobic polymer and needs to be surrounded by the CTAB surfactant to have stability in water. In fact, it gives the NPs a highly positive potential and offers superb colloidal stability in water. All-purpose NPs present positive charges leading to a strong electrostatic interaction between CCPNs and the membrane of bacteria can occur, which provides an important basis for CCPNs to detect the presence of microbial cells and further break their membranes [22]. The stability of NPs CTAB/P3HT NPs dispersion in an aqueous solution was checked after six months of the fabrication and the same behavior, regarding the shape and size of NPs, was obtained, demonstrating the high stability of the NPs.

#### 3.2.2. Optical and Electrochemical Characterization

**Figure 2:**
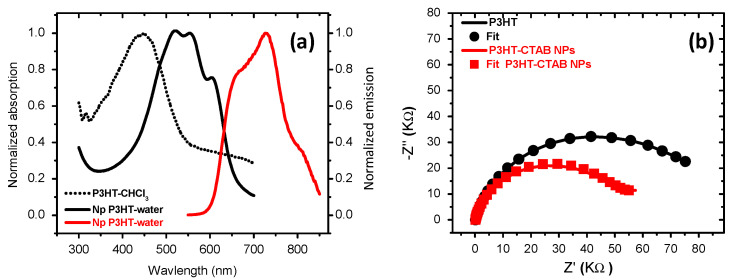
(**a**) Normalized absorption of P3HT (dashed line) and P3HT-CTAB NPs (black line), and emission spectra of P3HT-CTAB NPs (red line), (**b**) EIS of P3HT/GCE (black line) and CTAB-P3HT NPs/GCE (red line) obtained at potential of −0.4 V with DC of 10 mV and frequency range of 100 kHz to 0.1 Hz.

CTAB-P3HT NPs were characterized by UV/vis absorption and fluorescence spectroscopies. Figure 2a (dashed curve) shows the normalized absorption of P3HT in solution in chloroform with a maximum absorption at 448 nm. The curve of CTAB-P3HT NPs (black line) shows an absorption maximum at 520 nm, followed by shoulders at 560 nm and 610 nm with a tail extending up to 700 nm. The absorption of CTAB-P3HT NPs is red-shifted, Δλ = 72 nm compared to the P3HT, the maximum wavelength of absorption (λ_max_) of the nanoparticles is 520 nm, which is red-shifted for to the value determined in solution (λ_max_) 448 nm. Such an effect is well-known from studies in both thin-film and solid-state P3HT. The absorption spectra of CTAB-P3HT NPs are formed of two parts: a high energy absorption attributed to the amorphous phase of P3HT and a low energy region that carries the vibronic structure of P3HT aggregated within NPs [39]. This elucidates that P3HT chains cannot be dissolved in an aqueous solution and are encapsulated into a droplet in the aqueous surfactant solution which means that the absorption of the amorphous phase is due to un-aggregated chains within the nanoparticles and not from free P3HT chains in the suspension. When excited at their respective absorption peak wavelengths, strong fluorescence from the P3HT was observed with their emission maxima around 750 nm (red line) which should have a modest fluorescence quantum yield of Φ = 0.07 [43]. Electrochemical characterization was performed using EIS in a PBS solution without any additional external redox probe, to investigate the electrical properties of the electroactive layer. The potential investigated was −0.4 V vs. Ag/AgCl where the polymer was accordingly in its semiconducting state and a semicircle was obtained. Figure 2b shows the recorded Nyquist plots for films of P3HT/GCE and of CTAB-P3HT/GCE, the semicircle diameter is related to the charge transfer resistance of the electronic transfer from the polymer to the electrode. The fitting data (Table 2) from the equivalent circuit model illustrate the electronic properties of the layers and are represented by a charge transfer resistance (Rct) in parallel with a constant phase element (CPE), in series with the resistance of the solution (Rs). A Warburg impedance is observed in the case of the CTAB-P3HT NPs/GCE which is related to the diffusion of ions and demonstrates that the coating film from aqueous suspensions of CTAB-P3HT facilitates ionic insertion into the solution. The Rct value of the CTAB-P3HT film decreases compared to that of P3HT, which proves the improvement of ionic and electronic transfers.

### 3.3. Microbial Cell Biosensing

#### 3.3.1. Fluorescence Detection

**Figure 3:**
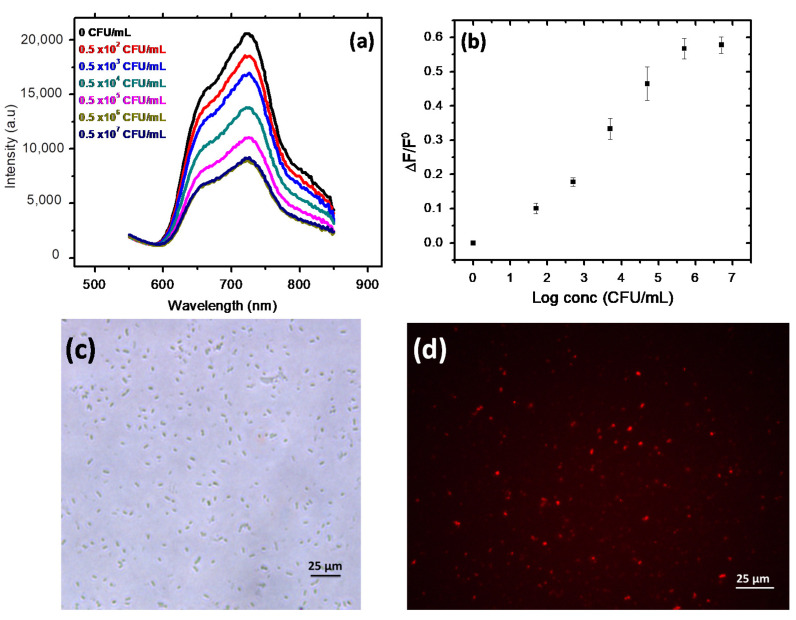
(**a**) *E. coli* detection measured by fluorescence spectra intensity (arbitrary units) in function of the wavelength (**b**) calibration curve [Correlation between the Log concentrations of *E. coli* loads with the average variation of ΔF/F^0^], (**c**) Phase-contrast image and (**d**) fluorescence image of *E. coli* incubated with CTAB-P3HT NPs, magnification of objective lens is X40.

As briefly mentioned in the introduction, the cationic conjugated polymer could electrostatically attract negatively charged particles [22,30] and lead to a decrease or chromic shift of its initial fluorescence [44]. Fluorescence sensing experiments were conducted at different concentrations of *E. coli* (0.5 × 10^2^–0.5 × 10^7^ CFU/mL) at a temperature of 22 °C. As shown in Figure 3a, the interactions between CTAB-P3HT NPs and intact microbial particles do not induce a fluorescence wavelength shift but rather a decrease in fluorescence intensity. The fluorescence quenching can be explained by the agglomeration of CTAB-P3HT NPs on *E. coli* due to the electrostatic interactions between cationic groups of CTAB-P3HT NPs and a highly negatively charged bacterial cell wall. This assay benefits from the simple “mix and detect” protocol and thus the fluorescence quenching strategy with a low signal from the background. Figure 3a shows the fluorescence spectra of CTAB-P3HT NPs for various *E. coli* concentrations. In pure water, a fluorescence peak maximum at 725 nm is observed. A significant decrease in fluorescence intensity was observed with a small concentration of 50 CFU/mL of *E. coli*. The drop in the fluorescence emission intensity continues with increasing *E. coli* concentration until 0.5 × 10^6^ CFU/mL was reached and after the signal stays stable for the higher concentration *E. coli* suggesting that all CATB-P3HT-NPs do not bind to bacteria. The calibration curve (Figure 3b) presents a relation between log concentration of bacterial cells and absolute change in fluorescence intensity (ΔF/F^0^) and results in a concentration-dependent quenching of the polymer fluorescence with a limit of detection (LOD) less than 5 CFU/mL. Since CATB-P3HT- are fluorescent NPs, fluorescence imagines were performed after incubation with model bacteria, *E. coli*. As shown in Figure 3d, the bacteria emit bright red fluorescence, indicating that NPs with positive charge agglomerate and bind tightly to bacteria via electrostatic interactions. Moreover, the zeta potential of *E. coli* was measured after incubation with CATB-P3HT-NPs. The zeta potential of *E. coli* was remarkably increased from −45.5 ± 5.59 mV to −16.5 ± 3.81 mV. These results agree with our assumption that CATB-P3HT-NPs are positively charged and agglomerate with bacteria through electrostatic interactions. The conventional fluorescence sensing approach of microbial pathogens involves ligand-receptor interactions. For example, Disney et al. reported carbohydrate functionalized CPs which recognize *E. coli* by multivalent interactions [45]. Nonetheless, non-specific detection based on electrostatic and hydrophobic interactions has been used to detect pathogens. Plante et al. [44] and Panda et al. [46] have reported fluorescence quenching upon binding of a cationic polythiophene derivative and gold nanoparticle-polythiophene composite to the cell surface of bacteria. However, the decrease in fluorescence intensity did not appear as significant as what we observed. To ensure that the fluorescence quenching is due to the electrostatic interaction between CTAB-P3HT NPs and the cell wall of *E. coli*, we prepared anionic P3HT NPs (SDS-P3HT NPs) which we used as a negative control. As shown in Figure S3, the fluorescence spectrum intensity of SDS-P3HT NPs did not show any change upon the addition of *E. coli*.

#### 3.3.2. Electrochemical Detection

**Figure 4:**
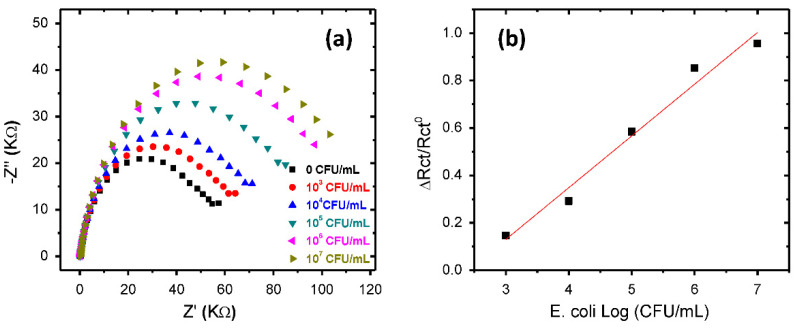
(**a**) Nyquist diagrams of a CTAB-P3HT NPs modified GCE obtained with increasing concentrations of *E. coli* in PBS and, (**b**) Calibration curve corresponding to average variation of ΔRct/Rct0 versus Log concentrations of *E. coli*.

The detection of *E. coli* via electrostatic interaction with CTAB-P3HT NPs-deposited on glassy carbon electrode (GCE) was investigated by EIS. In our previous study, we reported the detection and evaluation of *E. coli* by using EIS methods [47]. The sensing approach involved mannose *E. coli* pili protein recognition with a detection range from 10^3^ to 10^7^ CFU/mL, and high selectivity. Herein, we explore the use of the electrostatic interaction between CTAB-P3HT NPs and cell membranes without using any external redox indicator, thus the detection is directly proportional to the change in the electrical properties of the P3HT. Figure 4a shows the Nyquist plots of EIS for the GCE with different concentrations of *E. coli*, from10^3^ CFU/mL to 10^7^ CFU/mL. All modified electrodes show a large increase in diameter of the semicircle, after bacteria attachment, compared to that of the unmodified GCE, indicating much higher Rct values. This result highlights that the electrical properties of the sensing layers, such as charge transfer, resistance and capacitance, are affected by the attachment of bacteria. The Rct values after bacteria attachment were obtained from the equivalent circuit model and used to generate the calibration curve. The calibration curve shows a dynamic variation with the logarithm of bacterial concentration (Figure 4b), with a detection limit of 250 CFU/mL.

Table S1 shows an overall comparison of our results with those previously reported in the literature for the detection of foodborne bacteria [44,46,48]. These results show that CTAB-P3HT NPs have competitive sensing performance with an easy processability and high stability.

### 3.4. Antimicrobial Activity

Previous studies have reported dark mode antimicrobial activity of cationic NPs [41]. Consequently, exploring the role of the cationic group and hydrophobic π-conjugated system of NP on its antimicrobial activity, MIC, MBC, MFC and time-killing assay were studied in the dark against Gram-positive and Gram-negative bacteria as well as fungi. As shown in Table 3, the MIC results of CTAB-P3HT NPs against the tested bacteria in nutritive broth with a started concentration of 5 × 10^5^ CFU/mL were 2.5 μg/mL for both gram-positive and gram-negative bacteria. Simultaneously, an auspicious antifungal activity was recorded with MIC at 0.312 μg/mL against *C. albicans* with a started inoculum of 5 × 10^4^ CFU/mL. CTAB-P3HT NPs show MBC/MFC activity at similar concentrations as the observed MICs, which indicates that the NPs mainly have cidal activity and do not only statically immobilize the microorganisms. The strong electrostatic attraction of positively charged NPs to negatively charged microbes (see zeta potential measurements, Table 1) allows the destruction of cell walls without light induction. This is due to the synergistic activity of the conjugated polymer core combined with the cytotoxic effect of the QA head of CTAB molecules as one functional unit. Moreover, these small CCPNs could enter the bacterial cells and interact with the ionic contents of bacteria via electrostatic interactions causing irreversible microbial damage [41].

**Figure 5:**
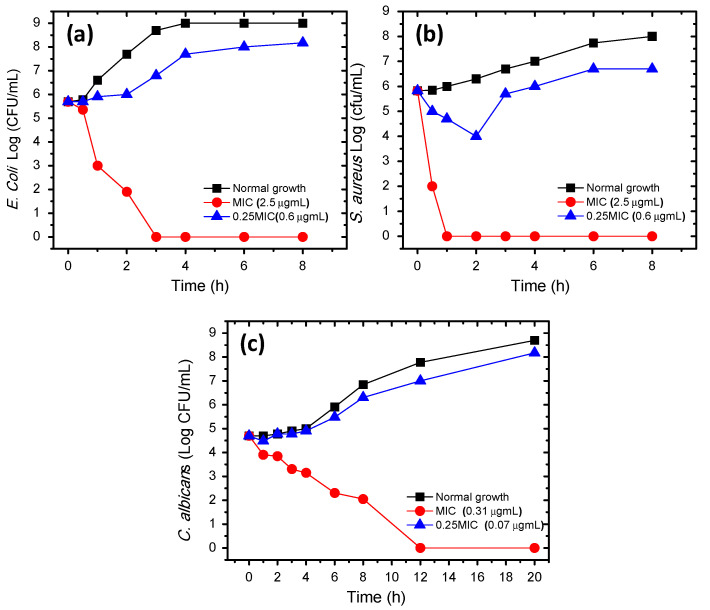
Time-killing curves for CTAB-P3HT NPs against (**a**) *E. coli*, (**b**) *S. aureus* and (**c**) *C. albicans* isolates. black line = Growth control, red line = MIC and blue line = 1/4 MIC.

To investigate the dynamic interaction between the antimicrobial CTAB-P3HT NPs and the microbial strain, time-killing patterns were studied (Figure 5). As shown in Figure 5a, 2.5 µg/mL (MIC) of CTAB-P3HT NPs has a stasis effect on *E. coli* for the first 30 min then kills 99% of *E. coli* in 1 h and 100% within 3 h. For 0.625 µg/mL (0.25 MIC), the stasis effect was observed for the first 2 h, then bacteria continued to grow normally, while in the case of *S. aureus* (Figure 5b), 2.5 µg/mL (MIC) of CTAB-P3HT NPs kill 99.9% in 30 min and 100% within 1 h. For 0.625 µg/mL (0.25 MIC), killing activity was observed at the beginning for CTAB-P3HT NPs then the survived bacteria revived, accommodated and continued to grow but at a slower rate than normal growth. In the case of *C. albicans* (Figure 5c), different effects are observed at a lower concentration. CTAB-P3HTNPs killed 100% of the fungi with a concentration of 0.312 µg/mL within 12 h while 0.078 µg/mL slightly decreased the rate of growth. This good effect on fungi at low concentration provides a powerful promising antifungal material. These results indicate that this kind of CCPN needs time to penetrate the cell wall and to cause destruction of the microbes, initially affecting the growth rate behavior of the microbe. Thus, *E. coli* has a very rapid growth rate and is the most resistant toward CCNPs, while *S. aureus* with a lower growth rate is less resistant and takes only 1 h for a 100% killing effect. In the case of *C. albicans*, with its slow growth rate, it takes more time to adapt showing a 4 h lag phase, which gives time for CCPNs to be attracted and to cause damage to fungal cells at a very low concentration (0.312 µg/mL) but within a longer time of about 12 h. Comparing these data to those previously reported in the literature with CTAB [49], we observe that CTAB could kill bacteria at high concentrations, which can produce severe cell cytotoxicity at that condition. Thus, combined CTAB-P3HT NPs exhibit high antibacterial activities at such low concentration and avoid using CTAB with high concentration that can damage normal cells and contaminate the environment.

### 3.5. Biocompatibility of CTAB-P3HT NPs

**Figure 6:**
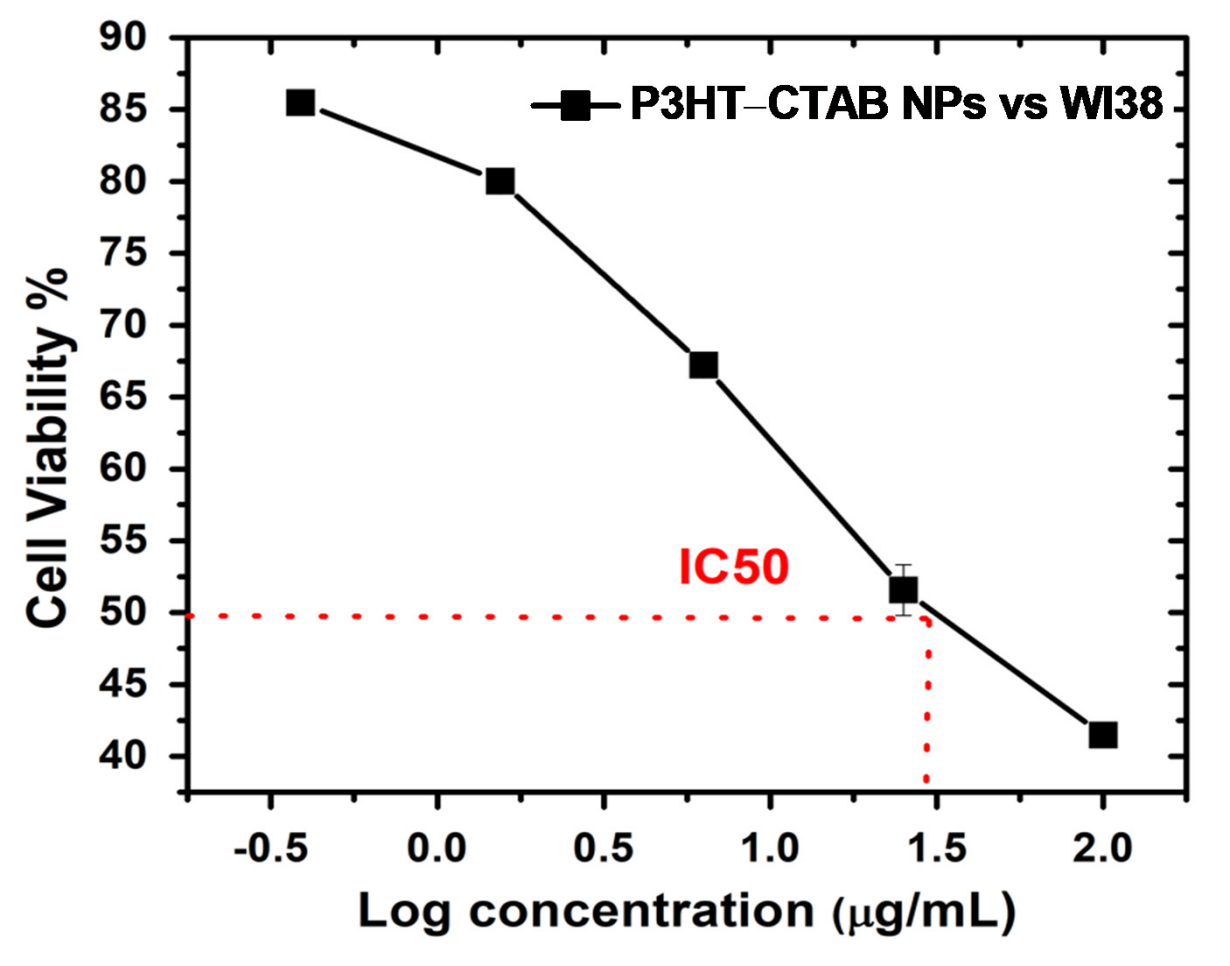
Cytotoxicity evaluation determined by MTT assay in Wl38 cells following the exposure to various concentrations of CTAB-P3HT NPs for 48 h.

To evaluate the cytotoxic activity of CTAB-P3HT NPs against normal cells, a typical MTT assay was used to study the biocompatibility of CCPNs at different doses (100 to 0.39 µg/mL) toward human diploid cell line (Wl38). Toxicity following 48 h of treatment with CTAB-P3HT is shown in Figure 6 revealing that cell viability was 85.2 ± 0.68, 80 ± 0.76, 67.21 ± 0.58, 51.55 ± 1.76 and 41.48 ± 0.47% following treatment with 0.39, 1.56, 6.25, 25 and 100 µg/mL of CTAB-P3HT NPs, respectively. Despite the observation that CTAB-P3HT NPs induced cell cytotoxicity in a concentration-dependent manner, the IC50 pattern of CTAB-P3HT was 27.69 µg/mL, which is a relatively high concentration in comparison to its bactericidal or its fungicidal activity. This suggests that CTAB-P3HT NPs are a novel biocompatible antimicrobial agent.

The selectivity obtained between bacteria and the mammalian cell is due to the significant differences in the superficial compositions and structures, including membrane phospholipid types, ratios and the peculiar cell walls of bacteria. In this study, we demonstrated that the CTAN-P3HT NPs target only pathogens such as bacteria and fungi but not mammalian cells. This obviously appears in the MTT test with mammalian cells after 48 h where biocompatibility is demonstrated compared to the cytotoxic effect obtained with bacterial or fungi. 

## 4. Conclusions

We have described a facile and cost-effective approach for preparing water-based cationic CP nanoparticles with high fluorescence and electrochemical properties. The combination of cationic surfactant CTAB to neutral CP (P3HT) provides positively charged nanoparticles that not only allow electrostatic interaction with bacteria and fungi for detection and quantification but also synergize the antimicrobial activity of conjugated polymers in the dark. The prepared CCPNs showed a direct microbial sensing system in solution by following the change in fluorescence properties and could be used as an active layer in electrochemical detection using EIS. Hence, the dual approach of detection was demonstrated. Furthermore, CTAB-P3HT NPs showed a broad spectrum of antibacterial and antifungal activity and without the requirement of light with good biocompatibility towards normal human cells. This work provides new biocompatible theragnostic nanoparticles for bacteria detection as well as for therapeutic treatment.

## Figures and Tables

**Table 1:** Zeta potential measurements of CTAB-P3HT NPs and microorganisms (*E. coli*, *S. aureus*, and *C. albicans*).

Name	Zeta Potential (mv)	SD
CTAB-P3HT NPs	+44.1 mv	±5.07 mv
*E. coli*	−45.5 mv	±5.59 mv
*S. aureus*	−39.3 mv	±4.81 mv
*C. albicans*	−32.8 mv	±4.54 mv

**Table 2:** Values obtained from equivalent circuit elements by fitting the EIS experimental data.

Element	Rs (Ω)	Rct (KΩ)	CPE (µF)	N	W1	X^2^
P3HT	172	79.2	4.2	0.85	----	0.019
CTAB-P3HT NPs	123	51.3	2.64	0.86	0.00014	0.012

**Table 3:** MIC and MBC/MFC of CTAB-P3HT NPs against three microbe isolates.

Pathogen	CTAB-P3HT NPs	
	MIC (µg/mL)	MBC/MFC(µg/mL)
*E. coli*	2.5	2.5
*S. aureus*	2.5	2.5
*C. albicans*	0.312	0.312

## Supplementary Materials

The following are available online at https://www.mdpi.com/1424-8220/21/5/1715/s1, Figure S1: ^1^H NMR spectra of P3HT in CDCl_3_, Figure S2: Fourier-transform infrared spectra of P3HT, and P3HT-CTAB NPs, Figure S3: The fluorescence spectra of P3HT-SDS NPs with different *E. coli* concentrations, Figure S4: Broth dilution method for determining MIC of P3HT-CTAB NPs with *E. coli* (a), *S. aureus* (b), and *C. albicans* (c), Table S1: Comparison of fluorescent conjugated polymer biosensors for the targeted detection and quantification of bacteria.
