# Supplementary material for: Antimicrobial Activity of Cationic Poly(3-hexylthiophene) Nanoparticles Coupled with Dual Fluorescent and Electrochemical Sensing: Theragnostic Prospect
Source: Sensors (Basel). 2021 Mar 2;21(5):1715. doi: 10.3390/s21051715 (PMC7958628; doi:10.3390/s21051715)
Supplement: Supplementary file 1 [file sensors-21-01715-s001.pdf]

Supplementary materials

Antimicrobial activity of cationic poly(3-hexylthiophene) nanoparticles coupled with dual fluorescent and electrochemical sensing: theragnostic prospect

Nada Elgiddawy[1, 2], Shiwei Ren[3], Wadih Ghattas[1], Waleed M. A. El Rouby[4], Ahmed O. El-Gendy[5,6], Ahmed A. Farghali[4], Abderrahim Yassar[3], Hafsa Korri-Youssoufi[1]*

[1] Université Paris-Saclay, CNRS, Institut de Chimie Moléculaire et des Matériaux d'Orsay (ICMMO), ECBB, 91400, Orsay, Ile-de-France, France, wadih.ghattas@universite-paris-saclay.fr

[2] Department of Biotechnology and Life Sciences, Faculty of Postgraduate Studies for Advanced Sciences (PSAS), Beni-Suef University, Beni-Suef, 62 511, Egypt; n.giddawy@psas.bsu.edu.eg

[3] LPICM, CNRS, Ecole Polytechnique, Institut Polytechnique de Paris, route de Saclay, 91128 Palaiseau, France; shiwei.ren@polytechnique.edu; abderrahim.yassar@polytechnique.edu

[4] Materials Science and Nanotechnology Department, Faculty of Postgraduate Studies for Advanced Sciences (PSAS), Beni-Suef University, Beni-Suef, 62 511, Egypt; waleedmohamedali@psas.bsu.edu.eg; ahmedfarghali74@yahoo.com

[5] Microbiology and Immunology Department, Faculty of Pharmacy, Beni-Suef University, Beni-Suef, Egypt

[6] Laser Institute for Research and Applications LIRA, Beni-Suef University, Beni-Suef 62511, Egypt; ahmed.elgendy@pharm.bsu.edu.eg

* Correspondence: hafsa.kori-youssoufi@universite-paris-saclay.fr

SI.1. Results and Discussion

SI.1.1. Characterization of the P3HT polymer

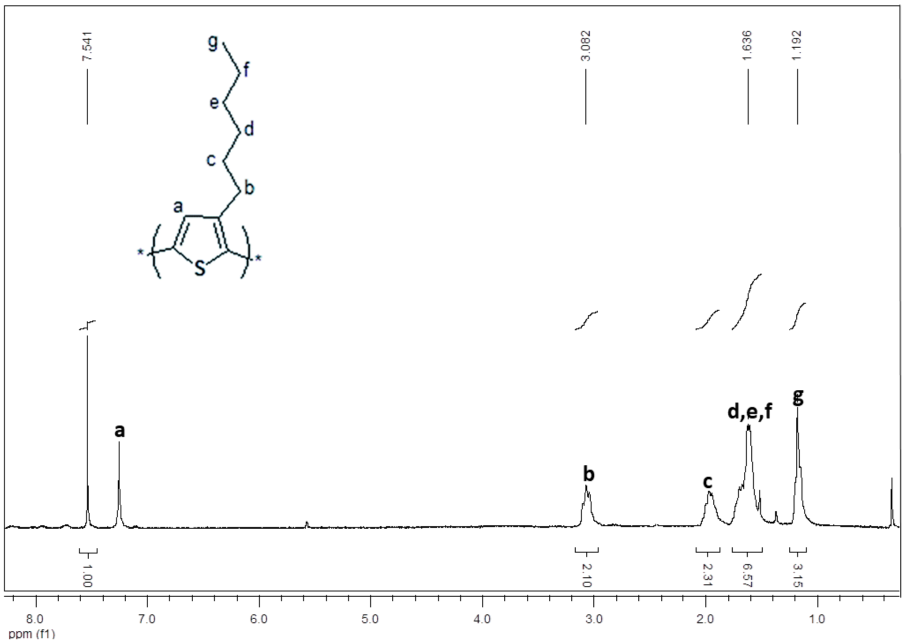

Figure S1. ^{1}H NMR spectra of P3HT in $CDCl_3$

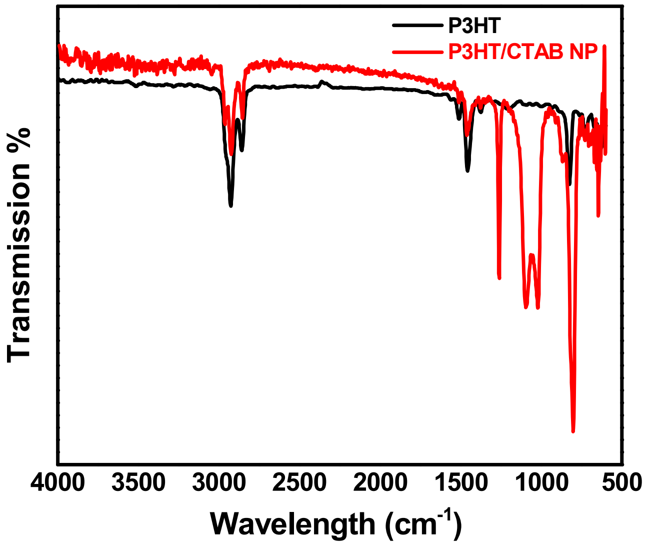

Figure S2. Fourier-transform infrared spectra of P3HT and P3HT-CTAB NPs

SI.1.2. Bacterial cell biosensing assay

SI.1.2.1. Fluorescence detection with opposite charge (Anionic conjugated polymer nanoparticles)

To explore the effect CTAB as cationic surfactant and to ensure that the diminution effect of polymer is due to the electrostatic attraction of nanoparticles to the cell wall of bacteria, Anionic conjugated polymer nanoparticles as ''negative control'' have been prepared by the opposite charge surfactant (SDS) by the same method , at its CMC which is (2 mg/ml). As shown in figure (S3), florescence spectrum intensity of NP prepared by opposite charge surfactant, SDS, shows No change in intensity in comparison with control (zero bacteria). This means that does not affected by any addition of bacteria.

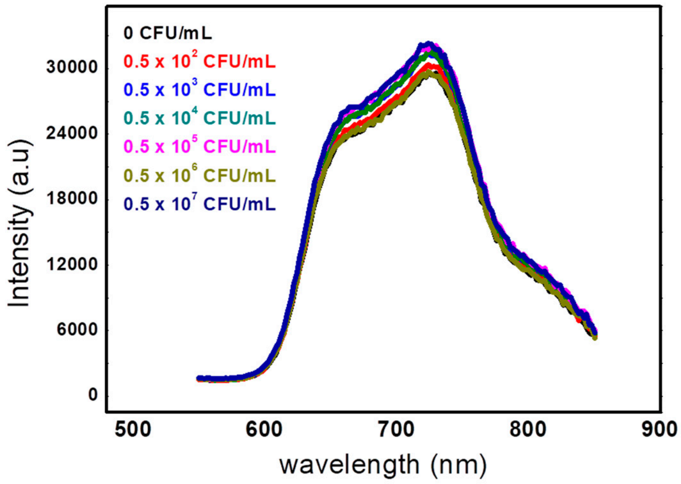

Figure S3. The fluorescence spectra of P3HT-SDS NPs with different *E. coli* concentrations

Table S1. Comparison of fluorescent conjugated polymer biosensors for the targeted detection and quantification of bacteria

Polymer	Detection method	Bacteria	LOD	Reference
AH-35 polythiophene biosensor	Fluorescence spectroscopy	*E. coli*	More than 500 CFU/mL	[1]
Au NPs–polythiophene composite	Fluorescence spectroscopy	*Gram-positive and Gram-negative bacteria*	1000 CFU/mL	[2]
PTP/TMP (complex of anionic conjugated PTP and cationic porphyrin (TMP))	FRET	*E. coli*	4.0 x 10^{4} CFU/mL	[3]
P3HT-CTAB NPs	Fluorescence spectroscopy	*E. coli*	5 CFU/mL	Our work
	EIS	*E. coli*	250 CFU/mL	

SI.1.3. Antimicrobial activity

SI.1.3.1. Minimum inhibitory concentration

(a)

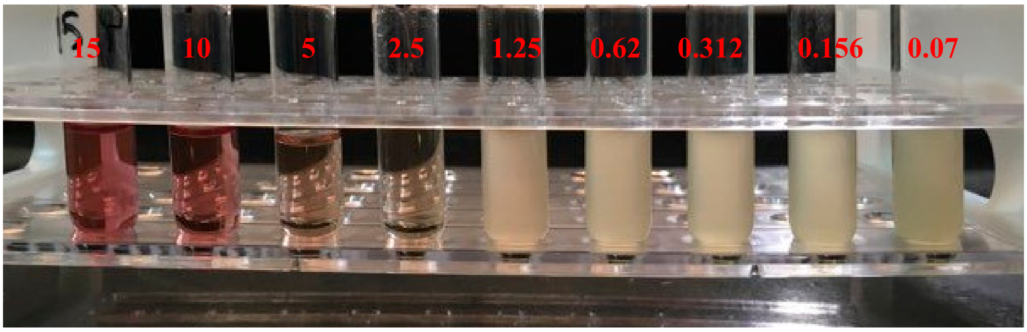

(b)

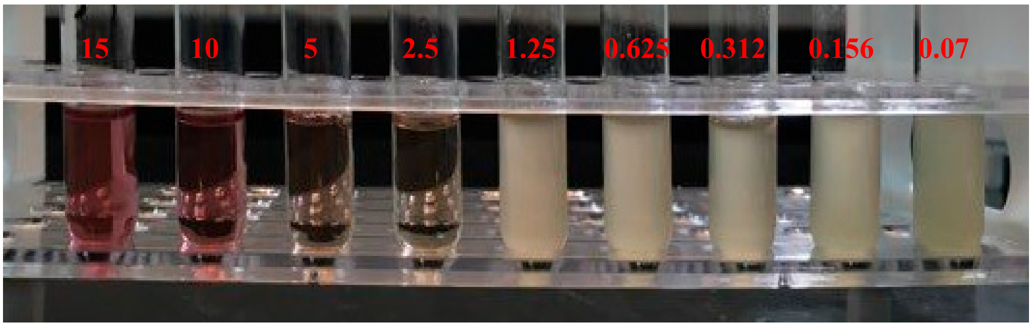

(c)

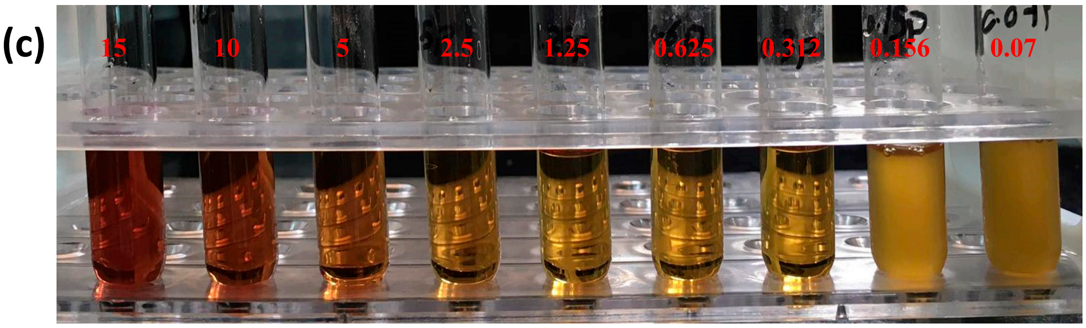

Figure S4. Broth dilution method for determining MIC of P3HT-CTAB NPs with *E. coli* (a), *S. aureus* (b), and *C. albicans* (c)

References:

1. Plante, Marie-Pier, et al. "Polythiophene biosensor for rapid detection of microbial particles in water." ACS applied materials & interfaces 5.11 (2013): 4544-4548.

2. Panda, Biswa Ranjan, et al. "Rapid estimation of bacteria by a fluorescent gold Nanoparticle–Polythiophene composite." Langmuir 24.20 (2008): 11995-12000.

3. Yan, Wenmin, et al. "Conjugated Polythiophene/Porphyrin Complex for Rapid and Simple Detection of Bacteria in Drinking Water." Macromolecular Chemistry and Physics 216.15 (2015): 1603-1608.
